# Predicted Aerobic Capacity of Asthmatic Children: A Research Study from Clinical Origin

**DOI:** 10.1155/2012/854652

**Published:** 2012-07-26

**Authors:** Lene Lochte

**Affiliations:** Centre for Child and Adolescent Health, Section of Aetiological Epidemiology, School of Social and Community Medicine, Faculty of Medicine and Dentistry, University of Bristol, Bristol BS8 2BN, UK

## Abstract

*Objective*. To compare longitudinally PAC of asthmatic children against that of healthy controls during ten months. *Methods*. Twenty-eight asthmatic children aged 7–15 years and 27 matched controls each performed six submaximal exercise tests on treadmill, which included a test of EIA (exercise-induced asthma). Predicted aerobic capacity (mLO_2_/min/kg) was calculated. Spirometry and development were measured. Physical activity, medication, and “ever asthma/current asthma” were reported by questionnaire. *Results*. Predicted aerobic capacity of asthmatics was lower than that of controls (*P* = 0.0015) across observation times and for both groups an important increase in predicted aerobic capacity according to time was observed (*P* < 0.001). FEV_1_ of the asthmatic children was within normal range. The majority (86%) of the asthmatics reported pulmonary symptoms to accompany their physical activity. Physical activity (hours per week) showed important effects for the variation in predicted aerobic capacity at baseline (*F* = 2.28, *P* = 0.061) and at the T4 observation (*F* = 3.03, *P* = 0.027) and the analyses showed important asthma/control group effects at baseline, month four, and month ten. Physical activity of the asthmatics correlated positively with predicted aerobic capacity. *Conclusion*. The asthmatic children had consistently low PAC when observed across time. Physical activity was positively associated with PAC in the asthmatics.

## 1. Introduction

Children with asthma often experienced breathlessness during physical activity and therefore tended to avoid vigorous physical activity with disadvantageous consequences to their physical conditioning [1, 2].

There are few paediatric pulmonary conditions in which physical activity has had such potentially harmful effect on patients, not only by limiting exercise capability, but also by acting as a direct stimulus to the underlying pathophysiology [3]. Exercise-induced asthma (EIA) has been recognized as one major manifestation of untreated asthma [4] with physical activity acknowledged as a powerful trigger of asthmatic disease [3, 5].

Physical activity in paediatric asthma has been influenced by physical as well as psychosocial variables. The comprehensive psychosocial variables included attitudes towards exercise. Asthmatic children have demonstrated negative attitudes towards physical activity [6] to be influenced by the limitations that they experienced in safely and unrestrictedly to join physical activities [5].

The cardiopulmonary fitness of asthmatic children was often suboptimal. Some studies revealed lower predicted aerobic capacity (PAC) among asthmatic children than with healthy controls [7–9]; yet, most such observations were cross-sectional. Evidence of PAC in healthy children was well established whereas PAC of asthmatic youths was scarcely documented, in particular from longitudinal observation.

The author hypothesized that the asthmatic children would demonstrate a different PAC than that of their healthy peers when observed over time. Therefore, the aim of the present study was to longitudinally compare PAC of asthmatic children against that of healthy controls during ten months.

## 2. Materials and Methods

### 2.1. Subjects

Twenty-eight asthmatic children and 27 controls without asthma volunteered. Data were collected at pretest, baseline, EIA-test, and following one, four, and ten months (Table 1). Eligible controls at month four were *n* = 24. Data were collected from October 1998 through April 1999.

The asthmatic group was selected according to the diagnose bronchial asthma as defined by the British Thoracic Society [10] and the control group was matched on age (±1 year), sex, weight (±5 kg), and height (±5 cm). The body weight, mean (SD) of asthmatics and controls was 40.0 (17.9) and 38.4 (13.8), respectively. Standing height, mean (SD) of asthmatics and controls was 143.7 (16.1) and 146.7 (17.8), respectively.

The asthmatic children were outpatients at the Paediatric Asthma and Allergy Clinic of Copenhagen University Hospital, Gentofte, Denmark, and they were included consecutively (informed consent obtained from parents) at their first visit. The control children were recruited from school classes of the Capital Region of Denmark and informed consent obtained from parents of the controls. All subjects were Caucasians.

Both groups were free from pulmonary tract infections, that is, forced expiratory volume in one sec (FEV_1_) was >60% of the expected normal value estimated from height. Descriptive information on the study population is shown in Table 2.

### 2.2. Questionnaire

Baseline data on asthma, allergy, and physical activity were obtained by a standardised questionnaire, published elsewhere [7]. The questionnaire was dispatched to all children, was identical for asthma and controls, and was completed by the children assisted by their parents.

#### 2.2.1. Asthma and Allergy

The questions on “ever asthma” and “current asthma” have previously been applied in surveys of asthmatic child populations [11]. The questionnaire included questions on pulmonary and allergy symptoms from skin, eyes, nose, lungs and symptoms frequency (daily, weekly, monthly, and/or every half-year).

#### 2.2.2. Physical Activity

Scores of physical activity as reported by hours per week were adopted from validated standards [12] of the Health Behaviour in School-Aged Children (HBSC) study [13]. For hours (h) per week, six response categories were applied: (i) none, (ii) 0.5 h, (iii) 1 h, (iv) 2-3 hs, (v) 4–6 hs, (vi) ≥7 hs. Physical activity referred to vigorous leisure-time physical activity, exercise, or sports outside school hours equivalent to at least slow jogging that made the children sweat or become out of breath.

Eighty-six per cent (*n* = 24) of the asthmatic children reported pulmonary symptoms such as wheezing, dyspnoea, chest tightness, and cough related to exercise. Of these, 46% (*n* = 11) reported allergic reactions such as eczema, 38% (*n* = 9) rhinoconjunctivitis, and 25% (*n* = 6) both eczema and rhinoconjunctivitis. Hospital journals confirmed that one-fifth of the asthmatic children were atopic and 7% (*n* = 2) had undergone allergy test; 14% (*n* = 4) had family predisposition.

Classification of asthma severity made reference to modified national guidelines, published elsewhere [7], based on present symptoms, lung function (PEFR), and medical treatment. PEFR, peak expiratory flow rate, (L/min) was self-recorded “same day morning and evening measurements” over the course of two weeks using Wright's peak flow meter (Airmed, Harlow, United Kingdom). The best of three PEFR exhalations was used for calculation. From the PEFR measurements, the percentage predicted values of PEFR (PEFR pred) was estimated using standard normal values according to height [14].

One PEFR registration was not returned and one was excluded due to extreme value. Hence, the study population comprised 13 children with mild asthma (PEFR pred >90–100%) 11 children with moderate (PEFR pred = 80–90%), and 2 children suffered severe asthma (PEFR pred <80%).

### 2.3. Design

The study population was followed prospectively by treadmill exercise tests during ten months.

### 2.4. Procedures

The age of the children was calculated from the date of birth to the nearest 0.01 years. Each child performed one pretest of PAC to establish running speed (RS) at an individual level according to sex and age, and to preclude training effect [4]. The pretest served to familiarise the child with the clinical environment and procedures. The baseline PAC-test was performed one week after the pretest.

All tests were performed separately with each child in the Clinic during the afternoon, and the asthmatic children performed the EIA-test prior to the pollen season. All instructions given during tests used comparable standards for asthmatic and control children.

A basic exercise warm-up programme preceded each exercise test. No EIA occurred during warmup. As a safety precaution, blood saturation was monitored during each test in all subjects by finger electrode method using Nellcor Symphony N-3000-120 (Nellcor, Boulder, CO, USA).

Body weight (BW) was measured by a spring balance to the nearest 0.1 kg (indoor clothing worn). Standing height was measured by a stadiometer to the nearest 0.1 cm and no shoes were worn during the measurements. Body mass index (BMI) was calculated as BW (kg)/height^2^ (m). The anthropometric apparatus was calibrated according to the standards of the manufacturers.

The endocrinological development of children aged ≥10 years was assessed by three methods: (i) the presence of menarche in girls, (ii) sex characteristics (breast size in girls and pubic hair in girls and boys) evaluated according to standards [15] and (iii) the testicular volume of boys estimated by comparison with an ellipsoid of known volume using Prader Orchidometer, Zachmann, 1974, [15]. The results were presented in Table S1 (see Supplementary Material available online at doi:10.1155/2012/854652).

Majorities of all children measured were distributed at pubic hair stage 1-2 and breast stage 2 or beyond according to the standards [15]. The boys had testicular volumes developed to stage 1-2 in 67% (*n* = 6) and 71% (*n* = 5) of the evaluated asthmatics and controls, respectively.

The PAC-tests were conducted without influencing ongoing medical treatment (corticosteroids: budesonide; *β*_2_-agonist: terbutaline (short term); salmeterol and formoterol (long term).

Prior to the EIA-test the intake of *β*_2_-agonist was discontinued for short term (12 hours) and long term (24 hours). No controls received medication.

HR at rest (HR_rest_) was monitored following 30 min of rest in a horizontal position. Heart rate (HR (beats/min)) during the PAC and EIA-tests was continuously monitored in all children by a thoracic Polar Sport Tester band (Polar, Kempele, Finland).

FEV_1_ (mL) was measured in a standing position by a Vitalograf (Spiropharma, Vitalograf Gold Standard, Klampenborg, Denmark). FEV_1_ was measured immediately before, 3, 5, and 10 minutes posttest. At the EIA-test FEV_1_ was further measured at 15, 20, and 30 minutes posttest.

At least three forced expirations were performed and children were instructed to blow from maximal inspiration and exhale quickly, forcefully, and for as long as possible into the instrument. Forced expiratory manoeuvres were repeated until two measurements of FEV_1_ within 100 mL of each other were obtained. The largest FEV_1_ value was used for analysis and from the FEV_1_ measurements the percentage predicted values of FEV_1_ (FEV_1_ pred) were estimated using standard normal values according to height [14]. The accuracy of the vitalograf was verified weekly by a calibrated syringe (Spiropharma), corrected to body temperature, atmospheric pressure, and saturation with water vapour (BTPS). The vitalograf did not need adjustment during the test period.

#### 2.4.1. PAC-Test

The PAC-test constituted a submaximal, 5 min exercise test of continuous running on a treadmill (Spiropharma, Cardiogenics no. 2113, Klampenborg, Denmark). The test constituted continuous increments of RS for the first 2 min, and RS was adjusted to a submaximal HR of 170–180 beats per minute (bpm). When HR attained steady state (HR_steadystate_), the current speed was maintained for 3 min; HR_steadystate_ (bpm) and RS (km/h) were registered for the estimation of PAC (mLO_2_/min/kg).

#### 2.4.2. EIA-Test

The EIA-test followed standardised guidelines using continuous increments of RS and inclination of the treadmill (Spiropharma, Cardiogenics no. 2113, Klampenborg, Denmark) during 5 minutes. When a submaximal HR of 180–190 bpm was attained, RS was kept constant for the duration of the test. The range of inclination was 5–15% and each subject wore a nose clip.

The pulmonary response from the EIA-test was expressed as the maximal percentage decrease in FEV_1_ (ΔFEV_1_%) from baseline (baseline value—lowest recorded postexercise value/baseline value × 100%) [4]. This value was used to assess the occurrence of EIA with the cutoff value set at 15% [4, 16]. No asthmatic children reached the set cut-off. The mean (95% CI) ΔFEV_1_% was 3.8 (1.5; 6.2) and 1.3 (−1.1; 3.8) in asthma and controls, respectively.

#### 2.4.3. Equation PAC

PAC was estimated by the following variables: maximal heart rate, HR_max_ (220 bpm—age in years) (estimated), HR_steadystate_ *≈* 170–180 bpm (measured), and HR_rest_ (measured). The estimation used a constant for child resting metabolic rate, RMR, (4.2 mLO_2_/min/kg) [17], and VO_2_ (mLO_2_/min/kg). The estimation used laboratory derived VO_2_ corresponding to RS at HR_steadystate_ published earlier [2].

The applied equation (below) was a modification of the original equation proposed by Klausen et al. [18]. The calculation resulted in the estimation of PAC adjusted for BW as follows:

(1) PAC=RMR+HRmax⁡−HRrestHRsteady  state−HRrest×(VO2−RMR).

The ambient room temperature and relative humidity were measured before each exercise test by a portable thermohygrograph (Model SL (Sound Level) 435007). The average room temperature and relative humidity of the PAC tests were 19.6 ± 1.9°C and 62.9 ± 10.6%, respectively; for the EIA-test the average room temperature and relative humidity were 19.0 ± 1.0°C and 58.1 ± 5.0%, respectively.

### 2.5. Statistics

General linear multiple regression analysis was conducted to determine the adjusted effects on PAC (APL*plus, Scientific Time Sharing Corporation, Rockville, MD, USA).

Two-way analysis of variance “repeated measures” was applied to evaluate the variations in the mean values of PAC over time within and between the groups of asthma and control children (SAS, v.8.2 PROC MIXED, SAS Institute Inc., Cary, NC, USA).

Multiple linear regression models were applied to estimate the groups (asthma and controls) specific regression lines using STATA (v.11.2) (Stata Corp, College Station, TX, USA). Age and sex adjustments were performed.

The sample size was calculated to comply with the power estimation, sensitivity (1 – *β*) = 80% and specificity (*α*) = 0.95. The significance tests applied were twotailed and the significance level set at 5%.

### 2.6. Ethics

The study was approved by the local Scientific Ethics Committee of Copenhagen County (no. KA 98029 m), the Danish Data Protection Agency (no. 1998-1200-320), and the Institutional Review Board at the Paediatric Asthma and Allergy Clinic of Copenhagen University Hospital, Gentofte, Denmark. The study followed procedures in accordance with the Declaration of Helsinki (1964).

## 3. Results

**Figure 1:**
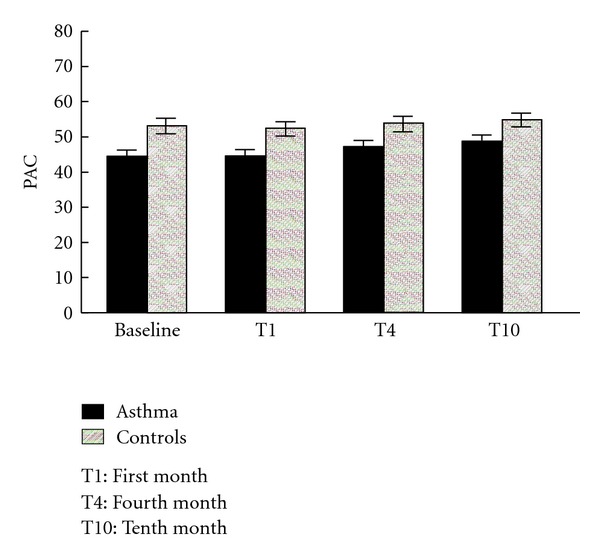
Mean PAC (mLO_2_/min/kg) with SE in the asthmatic and control groups of children according to four observation times.

Across the four times of observation, important group differences of PAC (*P* = 0.0015) between the asthmatic children and controls were seen with asthmatics depicting consistently lower PAC than controls. PAC of both groups of children increased gradually with time (*P *< 0.001) (Figure 1).

**Figure 2:**
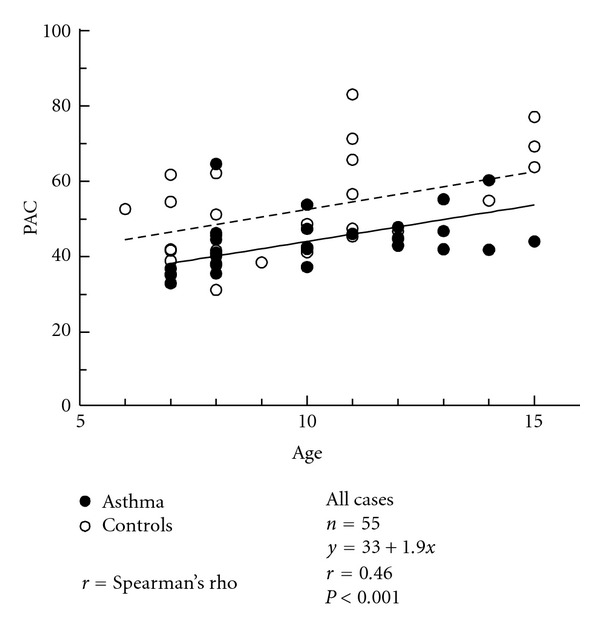
Baseline associations of PAC (mLO_2_/min/kg) by age (years) in asthmatic and control children.

PAC increased with age in the two groups of children. The baseline age effect (*F* = 14.73, *P* < 0.001) was illustrated in Figure 2. At baseline the asthmatics showed lower PAC values than controls (group effect: *F* = 10.35, *P* = 0.0020).

**Figure 3:**
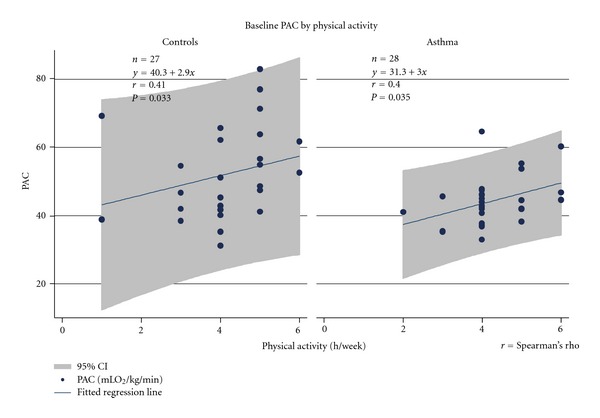
Baseline associations between PAC (mLO_2_/min/kg) and physical activity (h/week) in asthmatics and controls.

Figure 3 illustrated the associations between physical activity and PAC at baseline separate for asthmatics and controls. By analysing all children (*n* = 55) important effects of group (full and reduced models) and marginal effect of physical activity (full model) was found as follows: [(reduced model, effect of group: *F* = 7.29, *P* = 0.0093) (*R* ^2^ = 0.1209)], [(full model, effect of group: *F* = 5.52, *P *= 0.023; effect of physical activity (h/week): *F* = 2.28, *P* = 0.061); (*R* ^2^ = 0.2898, *P *= 0.0090)].

**Figure 4:**
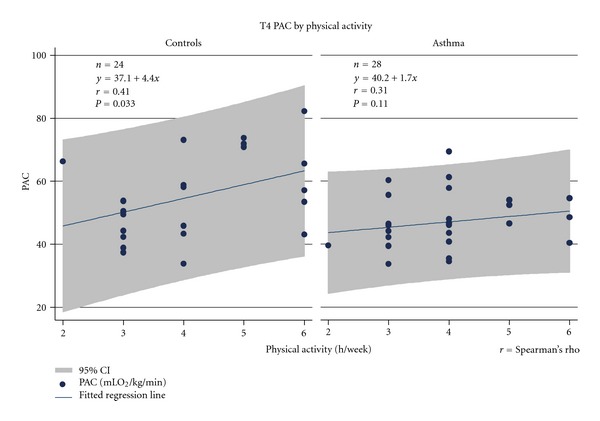
T4 associations between PAC (mLO_2_/min/kg) and physical activity (h/week) in asthmatics and controls.

Figure 4 illustrated the associations between physical activity and PAC at T4 separate for asthmatics and controls. Analysis of all children (*n* = 52) at T4 showed important effects of group and physical activity [(reduced model, effect of group: *F* = 6.86, *P* = 0.012; effect of physical activity (h/week): *F* = 3.03, *P* = 0.027); (*R* ^2^ = 0.3040, *P* = 0.0042)].

**Figure 5:**
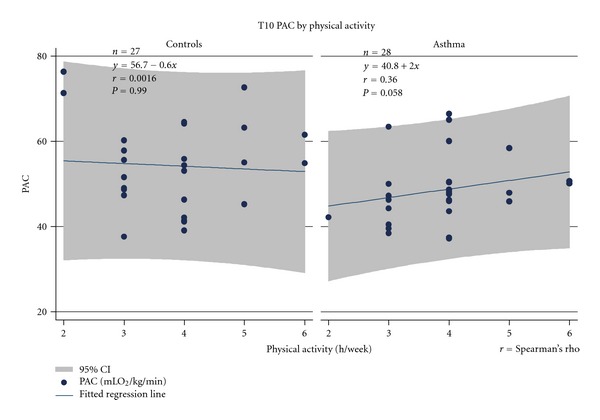
T10 associations between PAC (mLO_2_/min/kg) and physical activity (h/week) in asthmatics and controls.

Figure 5 illustrated the associations between physical activity and PAC at T10 separate for asthmatics and controls. By analysis of all children (*n* = 55) at T10 only the effect of group demonstrated importance [(reduced model, effect of group: *F* = 5.22, *P* = 0.026) (*R* ^2^ = 0.0897)].

Table S2 showed that in the asthmatic group, approximately 50% of children received medical treatment and the distribution was almost similar at all four time points.

Table S3 illustrated the distributions of “ever asthma” and “current asthma” for asthma and control children. Physical activity (h/week) did not show important effects (ns) for “ever asthma” or “current asthma” in asthmatics or controls (not illustrated).

## 4. Discussion

The study showed that PAC of the asthmatic children was lower than that of controls and differed between the four times of observation. During the ten months observation time, I found relative consistency in the importance of physical activity effects for PAC, and physical activity explained up to 30% of the variation in PAC. There were consistent and important group differences between asthmatics and controls.

This study was designed to limit well-known bias risks. Laboratory assessment of cardiopulmonary fitness has been documented to require considerable resources whereas the submaximal exercise test overcomes most of these limitations. The PAC-test represented a noninvasive and validated [2] submaximal method and was therefore preferred for the current study.

The requests for study participation followed consecutive order to ensure random selection of the asthmatic children. The procedure for selection of participants was closely monitored to ascertain sufficient time for the individual to contemplate participation. To avoid misclassification from, for example, disproportionate inclusion of highly motivated children (and parents), reflection time prior to giving consent was extended and further information provided as necessary.

The results showed that physical activity correlated positively with PAC, more consistently so for the asthmatics than controls, but physical activity did not influence “ever asthma” or “current asthma” and therefore these self-reports may not sustain the results. The reports of “ever asthma” and “current asthma,” nonetheless, complied with data from other Scandinavian child populations that used the same asthma and physical activity questions [19]. It cannot be excluded, however, that parents of the asthmatics who were well informed of benefits of physical activity for cardiopulmonary fitness in optimal asthma care, could have been overrepresented. Likewise, these parents may have recalled physical activity differently than other parents and could have overreported the physical activity levels of their children.

The study groups originated from slightly varying sociodemographic residential areas of the Capital Region. The variation could have influenced the data collection, for example, self-reports of physical activity in a nonrandom manner. Although unconfirmed, it was also possible that asthmatics and younger children received more support completing the questionnaire than controls.

The results on the endocrinological development of the included children were almost similar in asthmatics and controls. Although asthmatic PAC was consistently lower than that of controls, the stable increase of PAC by age in both groups may illustrate the well-recognized systematic influence of development on VO_2_ [20].

The participants with moderate and severe asthma who accounted for almost half of the asthmatic group may in fact have contributed disproportionately to the asthmatic reductions of PAC compared to the mild asthmatic subjects. Earlier findings from studies of severe childhood asthma [8] may lend support to this interpretation.

The asthmatic children demonstrated a similar increase of PAC over time to that seen for the healthy controls. It is plausible that this finding reflected a diminished asthmatic disease activity occurring during the observation time. However, this hypothesis needs to be prespecified and tested separately in future studies.

Indeed poor fitness of asthmatic children has been thoroughly investigated and physical risk factors documented [1, 3, 21, 22]. In healthy children, “previous” physical activity played an important role for the “present” activity level [23]. The reports of physical activity were collected at three different time points during ten months, but the current study was not designed to test for separate effects of physical activity in the asthmatics and controls investigated.

Over the course of the past decade, reviewers of asthmatic children's fitness have demonstrated a shift in their conclusions from previous “asthmatic children being less fit” [3] to current positions that “studies are inconclusive” [24]. This study complied with the former representing the times when data were collected.

The submaximal exercise test has been validated in different populations, and the validity of a submaximal exercise test depended on a predictable relationship between oxygen consumption and HR [25]. The equation applied in this study was validated under the assumption of a linear relationship between VO_2_ and HR and the submaximal method tended to underestimate PAC compared with laboratory monitored aerobic capacity [2]. Given such minor underestimation of the present PAC, there has not been an indication that this would have affected the groups in a differential way. Conversely, for the current full study population target heart rates were reached and hence the expected physiological stress levels.

The present results of the EIA-test indicated that lung function could have played a role in the asthmatic reduction of PAC. The decrease of the asthmatic lung function during the EIA-test potentially reflected the untreated nature of the asthmatic disease for half of the asthmatic children. Previous results of mild-to-moderate childhood asthma have shown that only sedentary children had impaired cardiopulmonary capacity [26]. The positive associations between physical activity and PAC that was seen for the asthmatics seemingly confirmed such results.

Earlier results published by my group found lower HR_steadystate_ of the control group than of the asthma group to suggest that physical activity could have influenced HR differently in asthmatic and nonasthmatic children [2]. Even though regressions slopes for HR and VO_2_ have been documented to alter with pulmonary disease [27], applying explanations like those to the present asthmatic PAC reductions would involve laboratory replication.

Although the present results have been generated from relatively small sample sizes, initial precautions were taken to power the study adequately. The longitudinal design served to demonstrate consistency of the results for PAC. While these data cannot ascertain causal associations between physical activity and PAC, they are nonetheless consistent with findings of others for both physical activity [19] and PAC [8, 9]. The reduced aerobic capacity of the asthmatic study group, consistent across observation times, may further add to the biological credibility of the current study hypothesis.

The extent to which the present asthmatic study population reported limitations from pulmonary symptoms disagreed with other asthmatic child populations studied [26]. Colleagues [26] investigated a large sample size and could report results of VO_2max_ from subgroups of sedentary and active (up to 2 hs/week) children that seemed comparable with the present results. The current sample, however, was not sufficiently sized to accommodate subgroup divisions. The frequent and largely untreated pulmonary symptoms reported to accompany physical activity by the asthmatic group could be a further explanation for the low asthmatic PAC that I found. Ultimately, the results may have been influenced by other known or unknown residual confounding factors that were not accounted for in this work.

BMI was higher than expected for the asthmatic group, which possibly indicated that the weight matching was not complete for the study population. VO_2_ was derived from established data on VO_2_ and running speed. Although baseline running speed showed no group differences, it cannot be excluded that the asthmatic children weighing slightly more than controls may have produced slower running speeds than controls at any one point during the ten months of observation time.

More recently, high BMI of asthmatic children and its relations with poor physical conditioning have been well acknowledged. Hence, the results suggested that rather than attempting to match participants on weight, future studies of asthmatic children might indeed generate important information from weight associations. In the light of the global weight burden with young people, the weight matching design may not be warranted when asthmatic children are under study.

In summary, the current results indicated that physical activity could be a risk factor for low PAC in the asthmatic children investigated. Although the results cannot necessarily be extrapolated to the global scene of asthmatic child populations, they underlined that joint monitoring of pulmonary symptoms and cardiopulmonary fitness of asthmatics may supply important information for prevention and treatment of those young suffering pulmonary symptoms by physical activity.

At a time when objective and individualised criteria to standardise exercise tests of asthmatic children are requested [3, 28], and where those that do exist have not been routinely implemented in paediatric asthma clinics, the public health implication of the current study is to target standardised monitoring of *pulmonary function* as well as * cardiopulmonary fitness* of asthmatic young; hence, to capture the variations of this burdening pulmonary childhood disease; the recommendations apply equally to Scandinavian and comparable populations of asthmatic children.

## 5. Conclusions

This study showed that the asthmatic children had consistently low PAC when observed across time. Physical activity was positively associated with PAC in the asthmatics.

## Figures and Tables

**Table 1:** Matrix of observations.

	Pretest	T0	T-EIA	T1	T4	T10
Age (year) and sex	X					
BMI	X				X	X
Development characteristics	X					
Ever asthma/current asthma	X					
FEV_1_		X	X	X	X	X
ΔFEV_1_			X			
HR_rest_					X	
Medical treatment		X	X	X	X	X
Menarche	X				X	X
PAC		X		X	X	X
PEFR					X	
Physical activity (h/week)		X			X	X
RS	X	X	X	X	X	X
Symptoms (allergic/pulmonary)	X					

T0: time point of baseline test, T-EIA: time point EIA-test, T1: time point 1st month test, T4: time point 4th month test, T10: time point 10th month test, BMI: body mass index (kg/m^2^), FEV_1_: forced expiratory volume in one second (mL), ΔFEV_1_: maximal percentage decrease in FEV_1_ from baseline, HR_rest_: heart rate at rest (beats/min), PAC: predicted aerobic capacity (mLO_2_/min/kg), PEFR: peak expiratory flow rate (L/min), and RS: running speed (km/h).

**Table 2:** Descriptive information, mean (±SD), of study population by group.

Baseline			
	Asthma	*P*	Control
	♂ / ♀		♂ / ♀
*N*	17 / 11		16 / 11
Age	10.1 (2.5)	ns	9.9 (2.7)
BMI	18.8 (3.8)	= 0.046	17.1 (2.0)
FEV_1_	2.2 (0.8)	ns	2.3 (1.0)
PAC	44.3 (7.3)	= 0.009	52.1 (13.4)
RS	7.5 (1.6)	ns	7.7 (1.3)

*N*: number

Age (years), BMI: body mass index

(kg/m^2^), FEV_1_: forced expiratory

volume in one second (L),

PAC: predicted aerobic capacity

(mLO_2_/min/kg),

RS: running speed (km/h)

*P*: probability, ns: nonsignificant.
